# Current therapeutic applications and pharmacokinetic modulations of ivermectin

**DOI:** 10.14202/vetworld.2019.1204-1211

**Published:** 2019-08-08

**Authors:** Khan Sharun, T. S. Shyamkumar, V. A. Aneesha, Kuldeep Dhama, Abhijit Motiram Pawde, Amar Pal

**Affiliations:** 1 Division of Surgery, ICAR-Indian Veterinary Research Institute, Bareilly, Uttar Pradesh, India; 2 Division of Pharmacology and Toxicology, ICAR-Indian Veterinary Research Institute, Bareilly, Uttar Pradesh, India; 3 Division of Pathology, ICAR-Indian Veterinary Research Institute, Bareilly, Uttar Pradesh, India

**Keywords:** ivermectin, ivermectin resistance, pharmacokinetic modulation, therapeutic applications

## Abstract

Ivermectin is considered to be a wonder drug due to its broad-spectrum antiparasitic activity against both ectoparasites and endoparasites (under class of endectocide) and has multiple applications in both veterinary and human medicine. In particular, ivermectin is commonly used in the treatment of different kinds of infections and infestations. By altering the vehicles used in the formulations, the pharmacokinetic properties of different ivermectin preparations can be altered. Since its development, various vehicles have been evaluated to assess the efficacy, safety, and therapeutic systemic concentrations of ivermectin in different species. A subcutaneous route of administration is preferred over a topical or an oral route for ivermectin due to superior bioavailability. Different formulations of ivermectin have been developed over the years, such as stabilized aqueous formulations, osmotic pumps, controlled release capsules, silicone carriers, zein microspheres, biodegradable microparticulate drug delivery systems, lipid nanocapsules, solid lipid nanoparticles, sustained-release ivermectin varnish, sustained-release ivermectin-loaded solid dispersion suspension, and biodegradable subcutaneous implants. However, several reports of ivermectin resistance have been identified in different parts of the world over the past few years. Continuous use of suboptimal formulations or sub-therapeutic plasma concentrations may predispose an individual to resistance toward ivermectin. The current research trend is focused toward the need for developing ivermectin formulations that are stable, effective, and safe and that reduce the number of doses required for complete clinical cure in different parasitic diseases. Therefore, single-dose long-acting preparations of ivermectin that provide effective therapeutic drug concentrations need to be developed and commercialized, which may revolutionize drug therapy and prophylaxis against various parasitic diseases in the near future. The present review highlights the current advances in pharmacokinetic modulation of ivermectin formulations and their potent therapeutic applications, issues related to emergence of ivermectin resistance, and future trends of ivermectin usage.

## Introduction

Ivermectin is a macrocyclic lactone obtained from the actinomycete, *Streptomyces avermitilis* [1]. It was the first macrocytic lactone anthelmintic to be introduced into veterinary use and is also the most widely used endectocide in animals [2]. It is considered a wonder drug, primarily due to its broad-spectrum antiparasitic activity against both ectoparasites and endoparasites in veterinary and human medicine. The activity spectrum of ivermectin is expanding every year making it one of the most useful drugs ever discovered.

Even though various antiparasitic drugs have lost their importance as therapeutic agents in managing parasitic diseases, ivermectin has been a popular drug of choice for the treatment of various parasitic diseases in humans and animals. Its mode of action includes opening of the glutamate-gated and gamma-aminobutyric acid-gated chloride channels, especially in invertebrates, thereby increasing the conductance of chloride ions leading to an increase in chloride transmission, which causes motor paralysis in parasites [3]. In a recent study, ivermectin was shown to potentiate glutamate-gated chloride channel receptors in invertebrate synapses by enhancing the amplitude of synaptic current and decay time. The study evaluated the glutamate-gated chloride channel receptors in the endoparasite *Haemonchus contortus* [4]. The characteristic feature of ivermectin is that its pharmacokinetic properties can be modified by altering the type of formulation [5]. The vehicle used in the pharmaceutical formulations of ivermectin plays an important role in absorption of drug from the injection site and thereby, its bioavailability [2].

The present review aimed to analyze the different pharmacokinetic modifications of ivermectin formulations that have been developed and utilized to ensure the wide spectrum activity of this drug in managing different parasitic diseases in veterinary and human medicine. Issues related to emergence of ivermectin resistance have also been presented.

## Therapeutic Applications Of Ivermectin

Since the development of ivermectin, it has been used to treat various diseases caused by ectoparasites and endoparasites in humans and other animals. However, ivermectin shows limited effectiveness against trematodes or cestodes [6]. It has high efficacy in the control of ectoparasites, such as fleas, flies, ticks, and mites that significantly affect weight gain and milk production in animals [7]. Ivermectin is administered on a monthly basis in canines as a prophylactic agent in controlling heartworm infection caused by *Dirofilaria immitis* [8].

**Figure-1:**
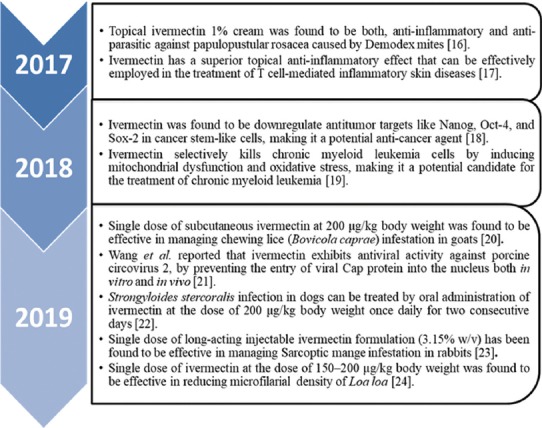
Chart representing the recent findings that extend the activity spectrum of ivermectin [16-24].

Ivermectin is also considered to be a safe therapeutic agent in human medicine, mainly in managing onchocerciasis. Furthermore, it is used as an effective microfilaricide at the recommended single dose of 150 µg/kg for up to 1 year [9]. It is also effective in eliminating microfilaria, *Wuchereria bancrofti* and *Brugia malayi* in humans [10]. Glaziou *et al*. reported that ivermectin is effective in treating head lice (*Pediculus capitis*) in human patients [11]. In a therapeutic trial conducted using oral ivermectin against human intestinal nematodes, it was found that this drug is effective against strongyloidiasis, ascariasis, trichuriasis, and enterobiasis [12]. Topical ivermectin preparation has been used for the safe and effective treatment of papulopustular rosacea (PPR) in humans [13]. PPR is a chronic cutaneous disorder characterized by persistent facial erythema and transient papules or pustules [14]. Recently, ivermectin gained importance as a new method of controlling malaria transmission in humans by targeting the zoophagic behavior of Anopheles mosquito that is responsible for its transmission. Administration of ivermectin in livestock was also found to be useful in tackling malaria transmission in humans by zoophagic vectors. Silicone-based slow release ivermectin implants when inserted in cattle caused mortality among the anopheles mosquito that feeds on the blood of cattle [15]. More recent applications of ivermectin are described in Figure-1[16-24].

## Impact Of Ivermectin Formulations On Its Pharmacokinetics

Pharmacokinetic parameters of ivermectin are influenced by various factors which include species, route of administration, vehicle used in the drug formulation, body weight, body condition, physiological status, and the nutritional status of the animal [25]. The most important factors that affect the bioavailability and duration of action of different ivermectin preparations include the route of administration and the type of formulations. Ivermectin is generally insoluble and unstable in aqueous preparations. Hence, to overcome the problem of poor water solubility and obtain a stable injectable formulation of ivermectin, several commercial preparations have been developed which use organic solvents as vehicles. However, high concentrations of organic solvents have been reported to induce significant side effects in many species [26]. Frosch reported that pain and inflammation at the injection site were commonly observed side effects of commercial ivermectin preparations [27]. The commercially available ivermectin preparations employ different vehicles that help in stabilizing the compound. It has been reported that the bioavailability of ivermectin varies according to the nature of vehicle [28]. Lifschitz *et al*. reported major differences in the pharmacokinetics of ivermectin in different generic preparations. These differences were significantly high with respect to the pattern of absorption from the site of injection and efficacy of the formulation as an antiparasitic agent [29]. Different formulations have been developed that facilitate different routes of administration and enable the development of different combinations with other antiparasitic drugs [30]. A time-dependent increase in the uptake of ivermectin was induced by oleic acid-containing complex micelles in an *in vitro* study conducted in Caco-2 cells [31]. In this study, oleic acid was found to be an efficient vehicle for ivermectin due to its ability to enhance the solubility and transport by reducing the p-glycoprotein (P-gp)-mediated efflux of ivermectin. The composition of various formulations of ivermectin and their characteristic features is described in Table-1 [7,26,28,32-61]. Vehicles, such as oils, liposomes, and other microparticles, can reduce drug metabolism and can enhance the release of large quantities of the active form of the drug over a long period of time to the target site by altering its pharmacokinetics [62].

**Table 1:** Different vehicles used in various ivermectin formulations in animals.

Composition of the formulation	Route of administration	Animal understudy	References
Stabilized aqueous formulation containing 0.1-7.5% w/v ivermectin (Parenteral administration) with other components such as surface active agent – 0.5-2.5% (polyoxyethylene sorbitan monoisostearate, polyoxyethylene sorbitan monostearate, and polysorbate 80). Cosolvent – 10-60% (glycerol formal, glycerin, and polyethylene glycol) and Substrate – 1-5% w/v (benzyl alcohol, lidocaine, parabens, and choline)	Parenteral and Oral	-	[32]
Oral administration of bolus containing ivermectin which is released by an osmotic pump	Oral	Cattle	[33]
Controlled release capsule administered orally using a specially designed balling gun which is formulated to deliver ivermectin for approximately 100 days at the rate of 1.6 mg/day	Oral	Sheep	[34,35]
Ivermectin is delivered using intraluminal controlled-release capsule	Intraluminal	Sheep	[36]
Subcutaneous and intramuscular administration of a novel oil-based formulation of ivermectin was found to be superior to the commercially available standard preparation	Subcutaneous and Intramuscular	Cattle	[37]
Formulation using silicone as a carrier that releases ivermectin over a long period of time. The lateral side of a cylindrical matrix-type formulation composed of ivermectin and silicone was used to produce a CR formulation	Subcutaneous	*In vitro* and *In vivo* (Mice)	[38]
Subcutaneous administration of ivermectin-loaded Poly (D, L-lactic-co-glycolic) acid microparticles was found to be an effective long-term ivermectin formulation	Subcutaneous	Dog	[39]
Zein microspheres 600 mg zein (plant protein isolated from corn) and 60 mg ivermectin were dissolved in 12 ml ethanol (66.7%). To this, 8 ml of ultrapure Milli-Q water was added and mixed using an agitator and tableted microspheres (Compressing 220 mg of microspheres containing ivermectin using a mold) were used for sustained-release of ivermectin	Oral	*In vitro*	[40]
Ivermectin was dissolved in a mixture of propylene glycol and glycerol formal at a ratio of 60:40 v/v that also contains 5% polyvinylpyrrolidone	Subcutaneous	Goat	[41]
Subcutaneous administration of ivermectin containing multilamellar liposomal vesicles made by distearoylphosphatidylcholine, cholesterol, and distearoylphosphatidyl-ethanolamine-polyethylene glycol5000 (DSPE-PEG5000) at the molar ratio of 1.85:1:0.15, respectively	Subcutaneous	Rabbit	[42]
Intravenous administration of ivermectin formulation containing propylene glycol: glycerol formal (60:40 v/v) containing 5% polyvinylpyrrolidone	Intravenous	Sheep	[28]
Commercially available ivermectin (3.15%) long-acting preparations (Ivomec Gold^®^, Merial) showed extended absorption process and long systemic persistence	Subcutaneous	Cattle	[43]
Sustained release solid dispersion was prepared by mixing ivermectin and hydrogenated castor oil which were further suspended in water to make an aqueous suspension that can be given subcutaneously	Subcutaneous	Sheep	[44]
Topical ivermectin formulations containing 1, 0.5, and 0.25% ivermectin were used that contains deionized water, olive oil USP, surfactants, shea butter, sorbitan tristearate, methylparaben, and propylparaben	Topical	*In vitro*	[45]
Ivermectin-loaded poly (lactide-co-glycolide) and poly (D, L-lactide) based microparticles were produced, which were used as sustained release parenteral ivermectin formulation	Parenteral	*In vitro*	[7]
*In situ* forming implants that acted as sustained-release formulation of ivermectin were prepared from biodegradable polymers such as poly (D, L-lactide) and biocompatible solvents such as N-methyl-2-pyrrolidone, 2-pyrrolidone, triacetin, and benzyl benzoate	Implants	*In vitro*	[46]
Fast-dissolving oral films containing ivermectin were administered orally. This method of oral drug delivery was found to be effective for long-term studies	Oral	Mice	[47]
Sterile biodegradable microparticulate drug delivery systems containing ivermectin which are based on PLA and PCL that can be used for subcutaneous administration	Subcutaneous	*In vitro*	[48]
Whole-body bathing method was used to deliver ivermectin to the skin without entering the plasma. The bath fluid contained ivermectin at a concentration of 100 ng/ml. This was found to be a more effective drug delivery system for the skin	Topical	Rat	[49]
Implant (silicone-CR formulation) is made up of two concentric silicone cylinders. The outer cylinder is a silicone impermeable membrane and the inner cylinder contains silicone along with a mixture of ivermectin, deoxycholate sodium, and sucrose	Subcutaneous	Rabbit	[50]
Ivermectin nanoemulsion (Cremophor EL^®^ -35-26 parts, Transcutol^®^ HP – 12 parts, ethyl oleate – 7 parts, ivermectin – 2 parts, and water – 53 parts) was evaluated for transdermal drug delivery and was found to be stable and effective in transdermal delivery of ivermectin	Transdermal	*In vitro*	[51]
Nanocarriers for the delivery of ivermectin using lipid nanocapsules which are prepared by a new phase inversion procedure	Subcutaneous	*In vitro* and *In vivo* (Wistar rats)	[52]
Ivermectin-loaded Soy phosphatidylcholine-sodium deoxycholate mixed micelles were administered subcutaneously to improve the aqueous solubility of ivermectin. They produced less local irritation when compared to commercially available preparations	Subcutaneous	Rabbit	[26]
Sustained-release ivermectin-loaded solid lipid dispersion was prepared in a lipid matrix of hydrogenated castor oil and was administered subcutaneously	Subcutaneous	Rabbit	[53]
SLNs were used as a vehicle for transdermal delivery of ivermectin. The SLNs were produced by hot homogenization combined with the ultrasonic method	Transdermal	*In vitro*	[54]
Sustained-release ivermectin varnish composed of 0.72 g of ivermectin, 3.6 g of amino methacrylate copolymer, 0.7 g of polyethylene glycol, and 2.15 g of hydroxypropyl cellulose per 100 ml of absolute ethanol	Topical	Zoo-housed animals	[55]
Sustained-release ivermectin-loaded solid dispersion suspension was formulated which was used in the therapeutic management of *Psoroptes cuniculi* infestation	Subcutaneous	Rabbit	[56]
Topical application of Palmitoyl-glycine-histidine gel spray formulations of ivermectin (0.1%), which was prepared from its aqueous solution by a heating and cooling method	Topical	Rat	[57]
Ivermectin bolus formulation containing 8% microcrystalline cellulose, 0.5% starch, and 0.25% low-substituted hydroxypropyl cellulose produced sustained-release of the drug for more than 60 days	Oral	*In vitro*	[58]
Ivermectin formulation containing self-emulsifying vehicles, such as sodium carboxymethylcellulose and poloxamers, was administered orally	Oral	Horse	[59]
Mixture of ivermectin and a-Tocopherol-loaded microparticles based on poly-D, L-lactide or poly-e-caprolactone together with sucrose and magnesium stearate were compressed to produce biodegradable subcutaneous implants	Subcutaneous implant	*In vitro*	[60]
Transdermal release of ivermectin using self-implanted tiny needles of hyaluronic acid encapsulated with ivermectin-poly (lactic-co-glycolic acid) microparticles	Transdermal implant	*In vitro* and *In vivo* (Rats)	[61]

CR=Covered-rod, SLNs=Solid lipid nanoparticles, PLA=Poly (D, L-lactide), PCL=Poly (ε-caprolactone)

Compared to the oral and topical application of ivermectin formulations, the subcutaneous route has been observed to be the most efficient route of administration in terms of bioavailability [63]. Following subcutaneous administration of ivermectin, the rate of absorption from the site of deposition and the rate of distribution and elimination of the drug were also found to be low [64]. Oral administration of ivermectin has relatively lower bioavailability due to binding of the drug with organic contents in the gut. In a study conducted in cattle, it was found that a large percentage of the orally administered ivermectin is excreted in feces [65].

Due to the immense potential of pharmacokinetic modulation of drug delivery for ivermectin, several formulations have been developed over the years that include stabilized aqueous formulations, osmotic pumps, controlled-release capsule, silicone carriers, zein microspheres, biodegradable microparticulate drug delivery systems, and biodegradable subcutaneous implants [32-35,38,40,48]. Several sustained-release formulations such as sustained-release ivermectin varnish and sustained-release ivermectin-loaded solid dispersion suspension were also developed during this period [7,44,46,53]. Advanced nanotechnology has also contributed to the development of formulations such as lipid nanocapsules and solid lipid nanoparticles that were found to be effective in modifying the pharmacokinetics of ivermectin [52,54].

## Emergence of Ivermectin Resistance

Ivermectin resistance has emerged as a major problem that limits its therapeutic uses. The mechanism of ivermectin resistance has not yet been established even though multiple hypotheses have been put forward by different researchers. Atif *et al*. reported that incorporation of endogenous ivermectin-insensitive subunits in the glutamate-gated chloride channel receptors in the synapses of the endoparasite *H. contortus* attenuated ivermectin action [66]. The G36A mutation that affects the third transmembrane domain of glutamate-gated chloride channel receptor was found to affect ivermectin sensitivity by decreasing the active period duration, thereby increasing receptor desensitization [4]. Genomic analysis of the ivermectin-resistant and sensitive isolates of the nematode *H. contortus* revealed that a single quantitative trait locus found on chromosome V was found to be linked with ivermectin resistance [67]. Shoop (1993) reported that most cases of ivermectin resistance resulted from the intensive use of ivermectin over several years [68].

Ivermectin resistance is detected by analysis of drug efficacy using methods such as post-mortem worm counts, nematode egg-count reduction, and *in vitro* assays for the development of immature stages of nematodes [69]. Lekimme *et al*. reported that the use of suboptimal formulations of ivermectin, such as pour-on and sustained release bolus products may cause emergence of resistance in Psoroptic mites [70]. Use of pour-on formulations of ivermectin results in relatively reduced bioavailability of this drug in horses. Such sub-therapeutic plasma concentrations of ivermectin may result in the development of drug resistance in parasites [2]. Xu *et al*. provided evidence that ivermectin resistance in nematodes can also be caused by alterations in P-gp and also pointed out that changes in P-gp in drug selected strains may contribute to ivermectin resistance *in vivo* [69]. Ivermectin resistance has also been reported in canine heartworm and *D. immitis* [71]. Ivermectin insensitivity is a major source of concern in intestinal infections by nematodes in goats, sheep, and cattle, which have a long history of intensive and long-term treatment with ivermectin [72]. Currie *et al*. reported the emergence of ivermectin resistance in *Sarcoptes scabiei* in patients treated with ivermectin for several years [73]. This projects the need for accurate and precise delivery of ivermectin into the systemic circulation, the failure of which is likely to result in the emergence of ivermectin resistance. At present, combination therapy is gaining popularity in the management of diseases that are resistant to conventional drugs. Ivermectin is combined with different therapeutic agents for improving the spectrum of activity against such parasitic diseases. Tribendimidine-ivermectin has been found to be a promising drug combination for the treatment of soil-transmitted helminthic infections [74].

## Conclusion and Future Prospects

In recent years, multiple efforts have been directed toward the development of long-acting formulations of ivermectin. The current trends in developing ivermectin formulations are aimed toward reducing the number of doses of the therapeutic agent required for complete clinical cure of different parasitic diseases. Such formulations will also provide prophylactic concentrations of ivermectin in systemic circulation for a longer duration so that recurrence of such parasitic diseases would be unlikely despite repeated exposure. Single-dose therapeutic protocols for the management of infection and infestations are also becoming popular. These can be applied effectively in case of lipophilic molecules, such as avermectin group of drugs.

By altering the composition of oil-based vehicles used in ivermectin formulation, researchers can program its release at therapeutic concentrations continuously for a long period of time. Long-acting formulations have the advantage of reducing toxicity and such preparations can be used safely either in species which are sensitive to the drug or those drugs which have a lesser safety margin. Nevertheless, one must also be careful, since incompetent formulations may result in sub-therapeutic levels that can eventually induce ivermectin resistance.

## Author’s Contributions

KS and TSS conceptualized the review, collected the literature and prepared the manuscript. VAA studied and edited the manuscript. KD, AMP, and AP carried out the proof reading and finalized the manuscript and guided entirely during the preparation of this manuscript. All authors read and approved the final manuscript.
